# From Tumor Cells to Endothelium and Gut Microbiome: A Complex Interaction Favoring the Metastasis Cascade

**DOI:** 10.3389/fonc.2022.804983

**Published:** 2022-05-05

**Authors:** Ali H. Abdel Sater, Youssef Bouferraa, Ghid Amhaz, Yolla Haibe, Ahmed El Lakkiss, Ali Shamseddine

**Affiliations:** Department of Internal Medicine, Division of Hematology/Oncology, American University of Beirut Medical Center, Beirut, Lebanon

**Keywords:** metastasis, endothelial cells, microbiota, immunotherapy, angiogenesis, gastrointestinal tract

## Abstract

Metastasis is a complicated process through which tumor cells disseminate to distant organs and adapt to novel tumor microenvironments. This multi-step cascade relies on the accumulation of genetic and epigenetic alterations within the tumor cells as well as the surrounding non-tumor stromal cells. Endothelial cells constitute a major player in promoting metastasis formation either by inducing the growth of tumor cells or by directing them towards dissemination in the blood or lymph. In fact, the direct and indirect interactions between tumor and endothelial cells were shown to activate several mechanisms allowing cancer cells’ invasion and extravasation. On the other side, gastrointestinal cancer development was shown to be associated with the disruption of the gut microbiome. While several proposed mechanisms have been investigated in this regard, gut and tumor-associated microbiota were shown to impact the gut endothelial barrier, increasing the dissemination of bacteria through the systemic circulation. This bacterial dislocation allows the formation of an inflammatory premetastatic niche in the distant organs promoting the metastatic cascade of primary tumors. In this review, we discuss the role of the endothelial cells in the metastatic cascade of tumors. We will focus on the role of the gut vascular barrier in the regulation metastasis. We will also discuss the interaction between this vascular barrier and the gut microbiota enhancing the process of metastasis. In addition, we will try to elucidate the different mechanisms through which this bacterial dislocation prepares the favorable metastatic niche at distant organs allowing the dissemination and successful deposition of tumor cells in the new microenvironments. Finally, and given the promising results of the studies combining immune checkpoint inhibitors with either microbiota alterations or anti-angiogenic therapy in many types of cancer, we will elaborate in this review the complex interaction between these 3 factors and their possible therapeutic combination to optimize response to treatment.

## Introduction

Throughout the course of history, cancer has proven to be a challenging and enigmatic disease that has burdened the human species (1). However, in the last three decades, our understanding of cancer has exponentially evolved regarding the nature of this malicious disease. Cancer is distinguished by a constant unregulated cellular proliferation which is mostly due to the activation of oncogenes and/or the inactivation of tumor suppressor genes (2). In their pivotal review ‘‘hallmarks of cancer’’, Hanahan and Weinberg aimed to establish the intricate nature of cancer into six main hallmarks: immortality, resistance to apoptosis, sustained angiogenesis, abnormal growth, tissue invasion and metastasis (3).

Metastasis is an intricate, stepwise process requiring the separation of tumor cells from the initial tumor site, the relocation to nearby structures and the migration *via* hematogenous spread to distant organs. Following the colonization of distant organs, cancer cells undergo proliferation and produce secondary tumors (4). Moreover, metastasis does not occur randomly; as such, colon cancer usually metastasizes to the liver, whereas prostate cancer metastasizes to the bones. However, few organs including the liver, lung and bone are common metastatic sites (5).

The most common types of gastrointestinal (GI) tumors are esophageal, gastric, colorectal, pancreatic and liver cancers (6). Less common types include neuroendocrine, anal, gallbladder and gastrointestinal stromal tumors (GIST) (7–9). A major factor implicated in cancer development is the tumor microenvironment (TME). TME plays a critical role in carcinogenesis. It incorporates diverse cellular components including proliferating cancerous, stromal, and endothelial cells (ECs); as well as fibroblasts and immune cells which are part of the adaptive immune system, such as lymphocytes, antigen presenting cells (APC), and B cells. In addition, cells of the innate immunity have been shown to partake in the TME, this includes monocytes, neutrophils, and natural killer cells (10–12). This interaction between cancerous and non-cancerous cells within the TME correlates with tumorigenesis (13). The non-cancerous cells have been shown to promote unregulated growth of tumor cells, whereas cancerous cells of the TME were involved in tissue invasion and metastasis (14, 15).

Another important factor involved in cancer progression and metastasis is the pre-metastatic niche (PMN). PMN refers to the microenvironment that facilitates tumor cell invasion and colonization of distant organ sites prior to the occurrence of metastatic spread (16). PMN emergence is a gradual process deriving from factors produced by tumor cells, including extracellular vesicles (EVs) and tumor-derived secreted factors (TDSFs) (17). These factors increase vascular permeability, modify extracellular matrix (ECM) and stromal cells, and alter the immune system (18–20).

Moreover, numerous studies have suggested a link between gut microbiota (GM) and the development of GI tumors (21, 22), especially concerning colorectal cancer (CRC) in which dysbiosis was associated with cancer progression (23). In addition, ECs appeared to play a major role in promoting cancer metastasis either by inducing tumor growth or by directing malignant cells towards blood or lymph for dissemination (24).

In this review, there will be exploration of the interaction between endothelial cells, gut vascular barrier, and the gut microbiota in the regulation of metastatic cascade of GI tumors and their enhancing effects. Moreover, the review will attempt to explain the different mechanisms through which bacterial dislocation promotes a favorable metastatic niche in distant organs allowing for dissemination and successful deposition of tumor cells in new microenvironments. To conclude, promising results found in previous studies that combined immune checkpoint inhibitors with either microbiota alterations or anti-angiogenic therapy give rise to a noteworthy discussion about their endothelial interactions and possible therapeutic combinations that potentially optimize treatment response in many cancers.

## The Pre-Metastatic Niche

Over the years, different theories have been proposed to explain the possible mechanisms behind metastasis. In 1889, the “seed and soil’ hypothesis was first introduced by Stephen Paget (25). The English surgeon noticed a non-random pattern of metastasis in the cancer of the breast. He also noticed a predominance of secondary growth in specific organs over others (25). Since then, Paget’s hypothesis has been challenged by many researchers and endorsed by others, with many questions remaining unanswered. In fact, in 1970, Isaiah Fidler demonstrated that despite the role of blood flow in metastasis, the latter can only occur at specific organ sites (26). It was also discovered that this organotropism in metastasis is independent of the vascular anatomy and/or the rate of blood flow to each organ (27). Moreover, it was determined that the sites of secondary seeding are majorly influenced by the microenvironment of the host tissue, in addition to the characteristics of the malignant cells (28).

The “seed and Soil” hypothesis established a solid ground that supported the emergence of the metastatic niche concept (29, 30). This concept suggests that circulating tumor cells (CTCs) from the circulatory system exit the circulation, and invade secondary organ sites to become disseminated tumor cells (DTCs) (29). This new host organ is called “the metastatic niche”.

In 2005, a study by Kaplan R. N. et al. presented the first proof of the existence of PMNs (31). Following injection of specific malignant cells of known metastatic potential in mice, analysis was conducted to monitor the fate of specific Bone marrow-derived cells (BMDCs). Through this study, it was revealed that BMDCs colonize the future metastatic sites before being invaded by the tumor cells (31). Hereafter, PMNs are pre-established microenvironments in distant organs, preconditioned at least in part by the primary tumors to promote the survival of CTCs before their arrival at these sites (18).

The formation of PMNs is complex and multifactorial. It is the net result of different tumor-dependent and tumor-independent pathological and physiological processes (30, 32). Different experimental studies were directed to understand these mechanisms of interactions. For this purpose, mouse models of experimental metastasis in organs like the liver, lungs, bone and lymph nodes were thoroughly investigated (18).

It is now established that the formation of PMNs starts with local changes: vascular leakiness and hyperpermeability being the first recognized step. This increase in permeability of blood vessels occurs in the sites of PMNs, and is induced by different factors (33, 34). Tumor-secreted factors like EGF receptor (EGFR) ligand epiregulin, cyclooxygenase 2 (COX2), members of the matrix metalloproteinase family (MMP) were demonstrated to play a role in the regulation and dysregulation of vascular barrier integrity in PMNs (35). Other factors, like transforming growth factor-β (TGFβ) act by inducing the expression of angiopoietin like 4 (ANGPTL4), consequently destabilizing ECs in future metastatic sites (36).

Furthermore, other stromal cell types are also affected by the action of the primary tumor. For example, fibroblasts induce the remodelling of the extracellular matrix (ECM) of PMNs (37). This remodelling is partly achieved by fibroblast-secreted enzymes that alter the existing ECM structure, and on the other part by the deposition of new ECM components (37). It was also found that, depending on the tumor cell type, specific S100 family members prompt the development of pro-inflammatory microenvironments that contribute to the formation of PMNs (38). For instance, breast cancer derived S100A4 cells resulted in the upregulation of Serum Amyloid A (SAA) proteins, like SAA1 and SAA2, which improved tumor cell adhesion to fibronectin and the recruitment of BMDCs to PMNs (39). Additionally, tissue-resident macrophages also play a role in supporting PMNs (40). Upon their activation by the primary breast tumor, pulmonary alveolar macrophages exert their effect by inhibiting the tumoricidal T helper 1 (TH1) cells, and tackling the proliferation and maturation of antigen-presenting dendritic cells, thereby contributing to immunosuppression and encouraging metastasis (40).

It is also important to further highlight on the role of other factors in the preparation of PMNs through ECM remodelling. After their accumulation in pre-metastatic organs such as the liver and the lungs, fibronectin enables the adhesion of BMDCs in these sites, hence, providing additional support for future metastasis (41). Additionally, Periostin, a protein secreted by stromal fibroblasts with α−smooth muscle actin and vimentin, leads to PMN formation through different mechanisms (42). First, it interacts intracellularly with ECM molecules, leading to the infiltration of metastasis-initiating cells through stimulating WNT signaling (42). Next, it is deposited outside the cell where it increases cell motility (42). Moreover, the immunosuppressive function of myeloid-derived suppressor cells (MDSCs) in the pre-metastatic lung of breast cancer was found to be affected by Periostin expression (43). Versican is another important factor implicated in the evolution of PMNs (44). It is a proteoglycan that can be derived from tumor cells, causing an emergence of an inflammatory microenvironment in the pre-metastatic lung (44). Parallelly, Versican can also be originated from CD11b+Ly6Chi myeloid cell present in the pre-metastatic lung (45). Finally, it was also proposed that the change in physical properties induced by ECM remodelling has a significant impact on disease progression (37, 46). As a matter of fact, collagen type I and type IV crosslinking, induced by a class of ECM-shaping enzymes, the lysyl oxidase family, was found to enhance the stiffness of tissues, thereby directly increasing tumor cell seeding, thus, promoting metastasis (37).

## Role of Endothelial Cells in Metastasis

Intravasation and extravasation are two fundamental principles of the metastasis cascade (47). They depend on the ability of cancer cells to cross the endothelial barrier, although approaching it from opposite sides (47). In other words, a disturbance of the endothelial junctions must occur so that malignant cells can disseminate into the bloodstream and invade distant organs. As such, it was proposed that altered ECs directly influence cancer inflammation and metastasis (48).

Intravasation is the act during which cancer cells can exit from the tissues to the circulation (48). It starts with tumor-induced angiogenesis, where new blood vessels with weak cell-cell junctions are formed (49). Moreover, different tumor-generated factors interact with ECs, affecting their function and promoting malignant dissemination. For example, TGFβ and vascular endothelial growth factor (VEGF) alter the endothelial barrier by increasing its permeability, and facilitating cancer cells’ intravasation (50). Also, the membrane MMP, expressed on breast cancer cells, act by disrupting the integrity of vessels surrounding the primary tumor, hence helping in the intravasation of malignant breast cells and assisting in their metastasis to the lungs (51). Likewise, invasive ductal carcinoma of the human breast was shown to express a disintegrin and metalloproteinase 12 (ADAM12) (52). This protein induces the shedding of ECs specific proteins (vascular endothelial cadherin and angioprotein 1 receptor TIE2) which also was suggested to impact the coherence of endothelial junctions, therefore helping in tumor intravasation (52).

Extravasation is when cancer cells leave the bloodstream to establish metastasis in different organ sites (47). The first step is the attachment and adhesion of disseminated tumor cells to ECs, and it usually take place in small capillaries (53). To extravasate, cancer cells must possess ligands and receptors that are compatible with those of ECs (eg. Integrins, cadherins, selectins, CD44 and immunoglobulin (Ig) superfamily receptors etc.) (47). Furthermore, chemokines secreted by stromal cells of distant organs play a role, not only in attracting cancer cells, but also in their adhesion and migration through ECs (54). In fact, an *in vitro* stimulation of CXC-chemokine ligand 12 contributed to the adhesion of prostate cancer cells to ECs, thereby increasing their transendothelial migration (TEM) (55). It was also proposed that altered endothelial barrier in the hyperpermeable PMN contribute to the process of extravasation (33). A study by Roblek et al. elaborated on the role of CCL2 in the metastatic process (56). Evidence was provided that CCL2 stimulation of endothelial cells altered the VE-cadherin/β-catenin complex, resulting in the loosening of the vascular endothelial barrier, and hence facilitating dissemination of cancer cells (56).

On the other hand, depending on their status, ECs may play an inhibitory or stimulating role in cancer progression. Factors secreted from quiescent ECs regulate inflammatory signaling and limit disease aggressiveness, while those released from dysfunctional ECs induce pro-inflammatory signaling, thus aggravating invasiveness and inducing metastasis (48). For example, Interleukin-6 secretion from ECs is increased when their integrity is disturbed, consequently stimulating metastasis (57). In contrast, the role of quiescent ECs in controlling inflammation was noted through balanced pathways of inhibitions, through IκBα for example, and activations, through NF-κB P65, leading to the inhibition of cancer progression and metastasis (57).

## Gut Microbiota and Implication in Metastasis

While the extent of research discussing the role of the microbiota in cancer has been exponentially increasing, the exact relationship between this microbial world and the pathogenesis of cancer remains not fully understood, in part because of the dual conflicting role in the promotion and inhibition of carcinogenesis (58, 59). However, it has been confirmed that the GM plays an essential role in the pathogenesis of several gastrointestinal cancers (60, 61). For example, *Helicobacter pylori* infection has been directly implicated in the development of gastric cancer (62). Moreover, *Fusobacterium nucleatum* was also associated with the development of colorectal cancer, and their DNA has been detected in the colorectal tumor cells (63). In fact, microbial pathogens have been shown to induce alterations in the host microenvironment that favor the transition of normal healthy cells into neoplastic cells (64). Several mechanisms have been proposed in this regard. These include but are not limited to dysbiosis, direct and indirect interactions with the immune system, induction of chronic inflammation, and molecular mimicry (61). Through those multiple mechanisms, the GM are able to influence the balance between immunosurveillance and carcinogenesis, favoring the development of neoplasms in multiple areas of the body, including the gastrointestinal tract (61).

**Figure 1:**
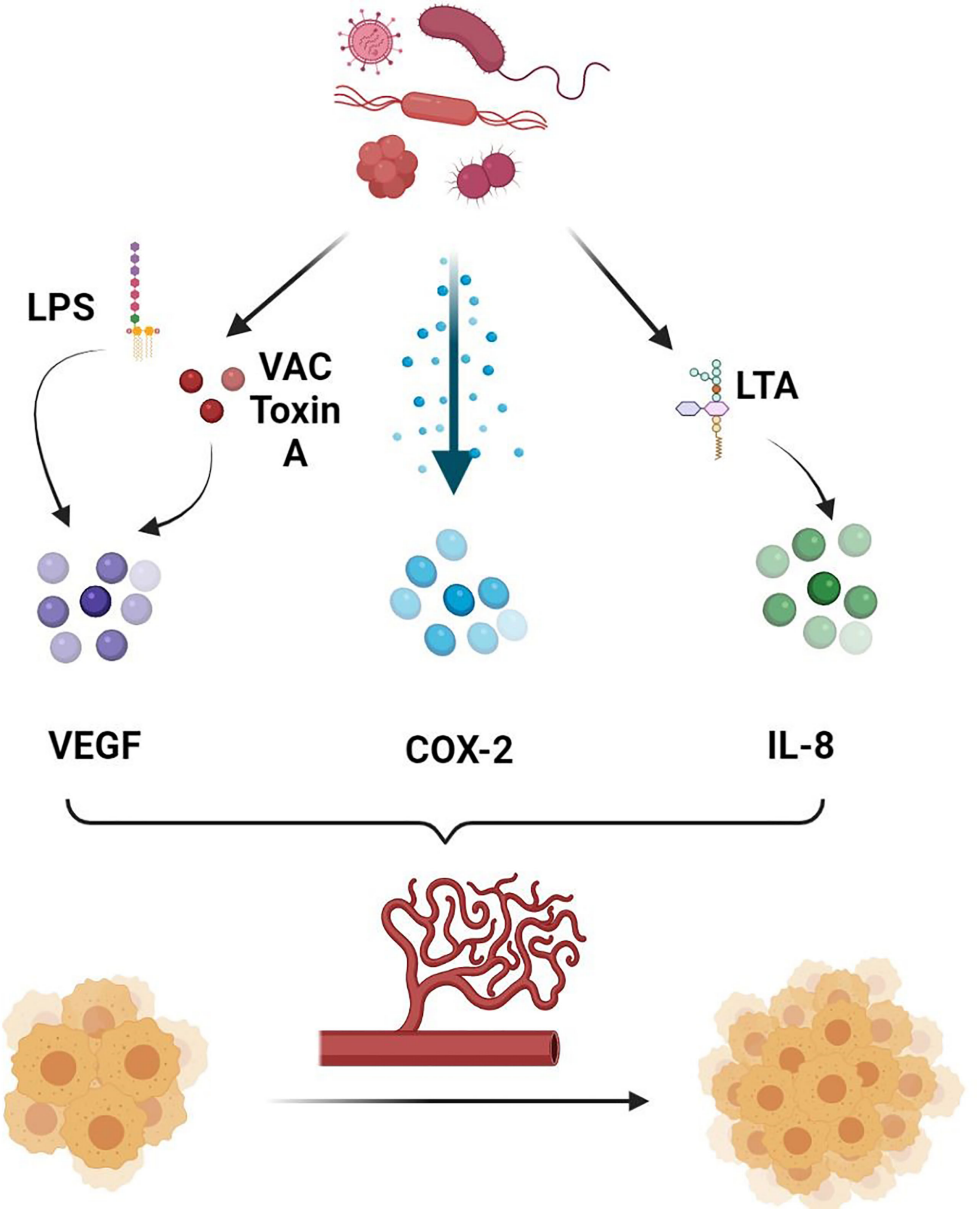
Role of gut microbiota in promoting angiogenesis. Gut microbiota secretes several molecules and chemokines in turn can induce the production of pro-angiogenic factors, allowing increased tumor angiogenesis and consequently tumor growth and metastasis. LPS, Lipopolysaccharide; VAC Toxin A, Vacuolating Toxin A; COX-2, Cyclooxygenase 2; LTA, Lipoteichoic Acid; IL-8, Interleukin 8.

In addition, the GM has been shown to directly and indirectly influence the ECs function and the process of angiogenesis and consequently facilitate the spread of neoplastic cells (65). They are known to release multiple metabolites that can promote angiogenesis (**Figure 1**). To start with, lipopolysaccharides (LPS) released by the gut microbial pathogens are able to induce the upregulation and activation of the VEGF (65). A study on pancreatic cancer has shown that the expression of VEGF and Toll-like receptor 4 (TLR-4) has been positively correlated with the micro-vessel density and the pro-angiogenic activity within the tumor microenvironment (TME), a process involving the activation of the PI3K/AKT pathway (66). In addition, vacuolating toxin A, one of the oncologic virulence factors produced by *H. pylori*, was shown to induce vacuolization and autophagy of gastric epithelial cells through several mechanisms, including the induction of VEGF secretion leading to angiogenesis and consequently carcinogenesis (67, 68). In fact, VEGF is well known to induce angiogenesis by exerting multiple effects on ECs (69, 70). First of all, VEGF has been shown to induce in-vitro ECs to invade the underlying matrix and form capillary-like tubules (70). Moreover, it was involved in induction of anti-apoptotic signals in ECs, allowing the survival of immature fragile vasculature (71). In addition, VEGF plays an essential role in the establishment of a vascular extracellular matrix allowing the growth of ECs (72). This is possible through the VEGF-induced increase in vascular permeability, allowing the leakage of proteins and other metabolites involved in the establishment of a nourishing extracellular matrix (72). Add to that the ability of VEGF to induce chemotaxis as well as the expression of collagenases and tissue plasminogen activator by ECs (70). As such, by inducing VEGF production, and through the mechanisms mentioned above, microbial metabolites including LPS and vacuolating toxin A will contribute to the increased formation and permeability of the vessels around the tumor cells favoring their metastatic dislocation.

In addition, lipoteichoic acid (LCA), another bacterial metabolite, has been shown to stimulate angiogenesis in colorectal cancer cells lines favoring their metastatic spread (73). This is possible through the simultaneous activation of extracellular signal-regulated kinases (ERK) 1/2 and the inhibition of the phosphorylation of the Signal transducer and activator of transcription (STAT) 3, increasing the expression of interleukin (IL) 8 (73). IL-8 is a potent pro-angiogenic chemokine and known to be highly expressed in colorectal cancer (74). Binding of IL-8 to CXCR-1 and CXCR-2 on ECs directly enhances ECs proliferation, MMP production, and consequently angiogenesis (75). In particular, by binding to CXCR-2 on human intestinal microvascular ECs, IL-8 increased their proliferation, chemotaxis and rapid stress fiber production (74). IL-8 was also shown to be elevated in gastric cancer, and its levels were higher in *H. pylori* infected as compared to non-infected samples (76). As such, targeting microbiota can potentially influence the angiogenic and metastatic activity of intestinal cancers, including colorectal cancer in particular. This is possible by decreasing the production of some bacterial metabolites such as LCA that can favor the metastasis of neoplastic cells through the mechanisms mentioned above.

COX-2 is another pro-inflammatory angiogenic marker that has been extensively studied in gastric cancer (77, 78). *H. pylori* has been shown to activate COX-2 by inducing DNA methylation/demethylation events, allowing tumor invasion and lymph node metastasis (77). Cytotoxin-associated genes (cag) were also associated with H. Pylori induced metastasis (79). In fact, it was shown that biliary cells expression of α5β1 integrin, a promotor of metastasis, was associated with the combined expression of cagA, cagL and cag pathogenicity island (cagPAI) (80, 81). Other studies also concluded that *H. pylori* can increase the fragility of ECs within the gastric cancer vessels through the secretion of biologically active proteins, including heat shock protein (HSP) 70 inhibitors (78). By doing so, *H. pylori* indirectly facilitates the spread of gastric cancer cells by increasing the fragility of the surrounding vasculature.

Moreover, *in vitro* studies have also proved the ability of GM to induce the activation of ECs leading to a specific angiogenic response in the gut (82). For instance, in a study by Schirbel et al, bacterial toxins specific for TLR-2/6 and 4 as well as nucleotide-binding oligomerization domain NOD 1 and 2 were able to induce proliferation, migration and tube formation by human intestinal microvascular ECs (HIMEC) (82). Although not yet demonstrated *in vivo*, those mechanisms may also be allowing direct influence of the GM on the tumor-associated vasculature that constitute an essential player in the metastatic spread of tumors.

On the other side, not all microbial metabolites were demonstrated to exacerbate the metastatic profile of cancers through a direct influence on the permeability, formation and proliferations of vessels. In fact, specific microbes were shown to have inhibitory effects on ECs’ proliferation, decreasing consequently angiogenesis and gastrointestinal cancers’ progression. The probiotic Prohep, made of a mixture of *Lactobacillus rhamnosus* GG, *E. coli* Nissle 1917, and heat inactivated VSL#3 (probiotic medical food [1:1:1]), was proven to decrease the extent of angiogenesis and inflammation in hepatocellular carcinoma (83). It allowed a shifting of the gut microbial population to specific species, including *Prevotella* and *Oscillibacter* (83). This leads to downregulation of Th17, decreasing therefore the production of IL-17, an angiogenic factor (83). In addition, enterotoxigenic E. coli (ETEC) produces a heat stable enterotoxin, that in turn activates a cGMP-dependent signaling pathway, leading to a decrease in VEGF and vascular cell adhesion molecule-1 (VCAM-1), both of which being closely related to angiogenesis and metastasis (84–86). Additional *in vitro* studies have also emphasized the inhibitory influence of microbes on angiogenesis. Pseudomonas aeruginosa secretes azurin and a corresponding peptide P28 that can penetrate human umbilical veins ECs (HUVECS), leading to the inhibition of VEGF- and basic fibroblast growth factor (FGF)-induced migration, tube formation and neo angiogenesis in several xenograft models (87). Bacillus anthracis was also involved in the inhibition of angiogenesis by blocking several pro-angiogenic pathways, including downregulation of VEGF and IL-8 (88, 89). As such, and through their inhibitory effects on some angiogenic molecules, some GM-associated metabolites can decrease the permeability and proliferation of blood vessels around the tumors decreasing their metastatic potential.

The relationship between bacteria and their human host can be pathogenic, neutral or beneficial (90). It may affect nutrients metabolism, protect from pathogen colonization, and manipulate immune response of host (91–93). A spectrum of diseases including cancer and immune disorders have been associated with gut microbiome disruption (94, 95). The intercellular communication between bacteria and their host is done through soluble products and membrane vesicles, known as bacterial extracellular vesicles (BEV) (96, 97). These vesicles carry enzymes, nucleic acids and toxins to be disseminated into the extracellular environment (98, 99). BEV are heterogeneous with different subtypes that vary based on their parent bacterium, structure, size, biological content, function, and formation paths and environmental growth conditions. Gram-negative and Gram-positive bacteria have different BEV structure and function as they follow different vesicle route formation. Gram-positive bacteria produce cytoplasmic membrane vesicles through bubbling cell death triggered by endolysin (100, 101). Gram-negative bacteria form different types of membrane vesicles that include outer membrane vesicles (OMV), outer-inner membrane vesicles (OIMV) and explosive outer membrane vesicles (EOMV). The formation of these vesicles happens through 2 pathways: the blebbing of outer membrane and the explosive cell lysis (97, 100).

BEV production rate and preference pathway are influenced by many factors including environmental composition, oxygen availability, temperature, chemical induced gene mutation and antibiotics exposure (100, 102). The membrane of gram negative and gram-positive BEV mirrors the membrane of parent bacteria from which it derived. The former contains lipopolysaccharides that engage with TLR4, and the latter shows surface lipoteichoic acids that interact with TLR2. OIMV, EOMV and CMV subtypes contain cytoplasmic (virulence factors, RNA and DNA) and membrane component (97, 100, 101).

BEVs are able to interact with host cells through engaging their microbe associated molecular pattern (MAMPs) or pathogen associated molecular patterns (PAMPs) with the host pattern recognition receptors (PRR) present on epithelial cells of mucosal surfaces and immune cells (103). The interaction between MAMPs/PAMPs and PRR can promote protective immunity, immune tolerance or even promote host pathology. It all depends on the parental bacterium from which the BEV was derived. For example, BEV originating from non-commensal bacteria may contribute to a worsening infection and may as well lead to sepsis (104–106). On the other hand, BEV originating from commensal bacteria may induce immunologic tolerance, hence provide protection from severe infections (107, 108).

Recently there has been a growing interest and consensus on the ability of BEV present in the gut lumen and derived from GM to bypass the epithelium and interact with macrophages, neutrophils and dendritic cells. Consequently, these interactions may encourage the access of BEV into the systemic circulation, leading to bacterial translocation and possible dissemination to the liver, lung and even to the brain (97, 109, 110). Additionally, the presence of BEV in the systemic circulation may also provoke different metabolic and immunologic responses in these organs (97, 109, 110). Several mechanisms have been described concerning BEV access to the systemic circulation. This access can occur through compromised integrity and function of intestinal epithelial barrier that changes its permeability by the action of different factors like diabetes, infection, inflammation, diet and caloric restriction (111). It can also be the result of active Trans-cellular migration through the non-compromised integrity of gut epithelium, or by the aid of dendritic cells and M-cells (112–114).

Tulkens et al. conducted a study where they were able to show the presence of circulating BEV probably originating from the gut or form microbial niches in other sites (111). This was done through detecting elevated levels of lipopolysaccharides positive BEVs in the plasma of patients with intestinal mucositis, inflammatory bowel disease and HIV who have compromised gut epithelial integrity due to gut microbiome disruption, known as microbial dysbiosis, compared to healthy individuals (111). The levels of circulating BEV positively correlated with plasma Zonulin level. The latter is responsible for the disassembly of the tight junction between gut epithelial cells by inducing phosphorylation of zonula occludens proteins, leading to an increase in intestinal barrier permeability, causing BEV translocation (111).

On the other hand, Jones et al. demonstrated that this translocation can as well happen in healthy individuals with intact epithelial barrier (115). The study described oral administration of fluorescent labeled BEV to mice, and a close follow-up to BEV distribution throughout the body. Most of orally ingested labeled BEV were found in the Gastrointestinal (GI) tract, whereas the rest were able to access the blood and lymphatic circulation, and reach different organ sites such as the liver, the heart and the lungs. This study was proof that BEV may cross cellular barriers in healthy individuals with intact epithelium, possibly by active trans-cellular migration to get access to distant organs (115). Furthermore, other studies reported the presence of nucleic acid from bacteria in the brain (116). This led to a speculation that these findings may be related to the presence of BEV in the systemic circulation and its ability to cross any host barrier including the blood brain barrier. Also, it was proposed that these bacterial nucleic acids may have been produced by brain resident bacteria (116).

Although the disruption of the gut microbiome was demonstrated to play a role in the GI tract oncogenesis and tumor progression, further studies are needed to better understand the mechanism behind it, how BEV affects different organs in healthy and sick individuals, and how it could influence the disease response to chemotherapy and immunotherapy (105, 117, 118).

## Discussion

### Complex Interaction Between GM, Checkpoints Inhibitors, and ECs and Their Possible Combination for Treatment

#### GM and CPIs

Immunotherapy, mainly through CPIs, constitutes a major advancement in the world of oncology (119). They mainly target the immune checkpoints like the programmed cell death 1 (PD-1), PD ligand 1 (PD-L1), and cytotoxic T-cell lymphocyte-associated protein (CTLA-4) (120). Their function lies in strictly controlling the T-cell immune system by manipulating the stimulatory and inhibitory proteins (121). Consequently, they contribute to the regulation of different systems including the activation of the cytotoxic T-lymphocyte, self-tolerance maintenance and autoimmunity prevention, as well as fine-tuning the duration and the intensity of the immune response to be able to avoid damaging the tissues in the period of inflammation (122–124). Cancerous cells tend to manipulate these checkpoints to be able to overcome the immune system and spread through the body (125). Multiple CPIs have been FDA approved throughout the time and play an important role, compared with chemotherapy, in prolonging the overall survival (OS) of patients with different malignancies within an accepted safety profile (122). With all the promising results of the CPIs, their success rate is only limited to a small population, which makes it a domain of interest to investigate the factors influencing the response in-order to be able to select the patients that could benefit from this treatment and consequently maximize its effects (125, 126).

One component identified is the high tumor burden (TMB), a crucial biomarker that could indicate enhanced response rate to CPIs (127). Adding to it the development in the genetic field where the identification of the defective DNA repair mechanism as well as the microsatellite instability burden (MSI) can increase the likelihood of benefit from immunotherapy (128). Furthermore, the tumor microenvironment can also contribute to the response to CPIs therapy, and that is by its ability to interfere with the specific and innate immune response, consequently influencing the growth of the tumor cells (129). The continuous changes in the energy metabolism, induced by cancer cells, affect immune cells in the TME. At some point, cancer cells consume the nutrients in the media and thus prevent effector T-cells activation. On the contrary, they can stimulate the regulatory immune cells through limiting their nutrition and thus resulting in CPIs resistance (130, 131). Other factors can include smoking, gender, BMI where all of these can alter the OS and progression free survival (PFS) of cancer patients (132–134). Recently, the GM has been emerging as an important element to study in evaluating the response to CPIs (135). With its role in immunosurveillance, the GM can positively influence the efficacy of CPIs (136–138). Multiple studies went on in the aim of proving this relationship between GM and CPIs (**Table 1**).

**Table 1:** Studies evaluating the relationship between GM and CPIs.

Study	Cancer type	GM status	Response to CPIs
Frankel et al. (138)	Metastatic melanoma	Presence of *Bacteroides caccae*	Increased response
Chaput et al. (139)		Enriched with *Faecalibacterium* and other *Fimicutes*	Increased OS and PFS
Salgia et al. (140)		Enhanced with *Ruminococcus obeum* and *Roseburia intestinalis*	Poor response
	Non-small-cell lung cancer (NSCLC)	Presence of *Lactobacilli and Clostridia*	Increase in the time to treatment failure
Matson et al. (141)		Enriched with *Alistipes putredinis*, *Bifidobacterium Longum* and *Prevotella copri*	Response to PD-1 blockade
		Enriched with *Ruminococcus* species	Attenuated response

Based on the provided evidence, it is now established that GM alter the response to CPIs, with the majority of studies confirming that the more diverse the GM the better the response. Nonetheless, it is important to establish a profound standardized profile to follow in this domain.

How is a local intestinal immune response initiated? Well, it usually starts by the recognition of the PAMPS through PRR like TLRs and Nucleoside-binding oligomeric domain proteins (NODs) (142). These PAMPs, through their interaction with PRRs, induce the growth of dendritic cells (DCs). Consequently, lymphocytes become activated and recruited to the site, augmenting the competence of antigen presentation (142). *Bifidobacteria* can perform the same job of promoting the growth of DCs maturation, but this requires a small concentration of antigens with higher sensitivity (143). DCs in their turn tend to boost the IFN-γ levels and consequently leads to the multiplication of specific CD8^+^ T cells boosting the antitumor effects with CPIs (144, 145). Furthermore, the efficacy of anti-CTLA-4 is enhanced through *B. fragilis* which tends to activate Th1 cells and their cross reactivity with bacterial antigens and new tumor antigens (146). It was also noted that the presence *of B. fragilis and B. cepacia* helped in decreasing the side effects of anti-CTLA-4 monoclonal Antibodies (147). This is explained by the capacity of *B. Fragilis* to enhance the proliferation of Treg as well as their ability to induce the conversion of CD4^+^ T cells to Tregs (148). In addition*, Akkermansia muciniphila* and *Enterococcus hirae* are linked to the presence of CD4^+^ central memory T cells (T_CMs_) in tumors which express C-X-C motif chemokine receptor 3 (CXCR3)/C-C motif chemokine receptor 9 (CCR9) (137, 149). The presence of these chemokines caused a prolongation of PFS and OS in advanced malignancies (150). Thus, through the activation of CD4^+^ T and CD8^+^ T in response to cross-reactivity of bacterial antigens, T cell employ anti-tumor effects (151). Moreover, intestinal *Faecalibacterium* also managed to induce DC maturation and consequently causing proliferation of CD4^+^ or CD8^+^ T cells enhancing the blockage of PD-1 (152).

#### GM and ECs

The endothelium is very important in the maintenance of homeostasis of the cardiovascular system as well as the functioning of the whole body (153, 154). It is protective against elevated blood pressures as well as atherosclerosis and it plays a crucial role in supporting the blood vessel as a barrier protecting the surrounding tissues from leukocytic infiltration and inflammatory processes (153, 154). The ECs are important in maintaining the physiological condition of the human system and that is by the production and release of different antiregulatory and anti-aggregatory mediators (153, 154).

The GM is made of different bacteria, protozoa, archaea, viruses and fungi, and it functions through a system of symbiosis among each other and with the human body (155). It is crucial for different human physiological conditions including digestion, immunomodulation, as well as cardiovascular system performance. Similarly, it influences different pathological conditions (155).

Gram-positive *Firmicutes*, Gram-negative *Bacteroidetes*, and Gram-positive *Actinobacteria* constitutes the healthy GM and their dysregulation, known as dysbiosis, is what leads to different gastrointestinal diseases like inflammatory bowel disease (IBD) and colorectal cancer (156). Dysbiosis can also contribute to different conditions like obesity, allergies, diabetes mellitus, and cardiovascular diseases (CVD) (157–159). After studying GM in CVD patients, it was noticed that patients with CVD have reduction in the beneficial bacterial diversity suggesting a direct link between both (160). GM contributes to the modulation of the immune system by altering the functionality of the neutrophils as well as T-cell differentiation into Th1, Th2, and Th17 or Treg (161). Moreover, through the fermentation of complex carbohydrates, GM secretes short-chain fatty acids (SCFAs) that cross the intestinal epithelium and alter the immune response (162). Thus, the balance between the GM institution and their metabolism was an approach to study in-order to reverse diet- and environment-induced vascular dysfunction (159).

**Figure 2:**
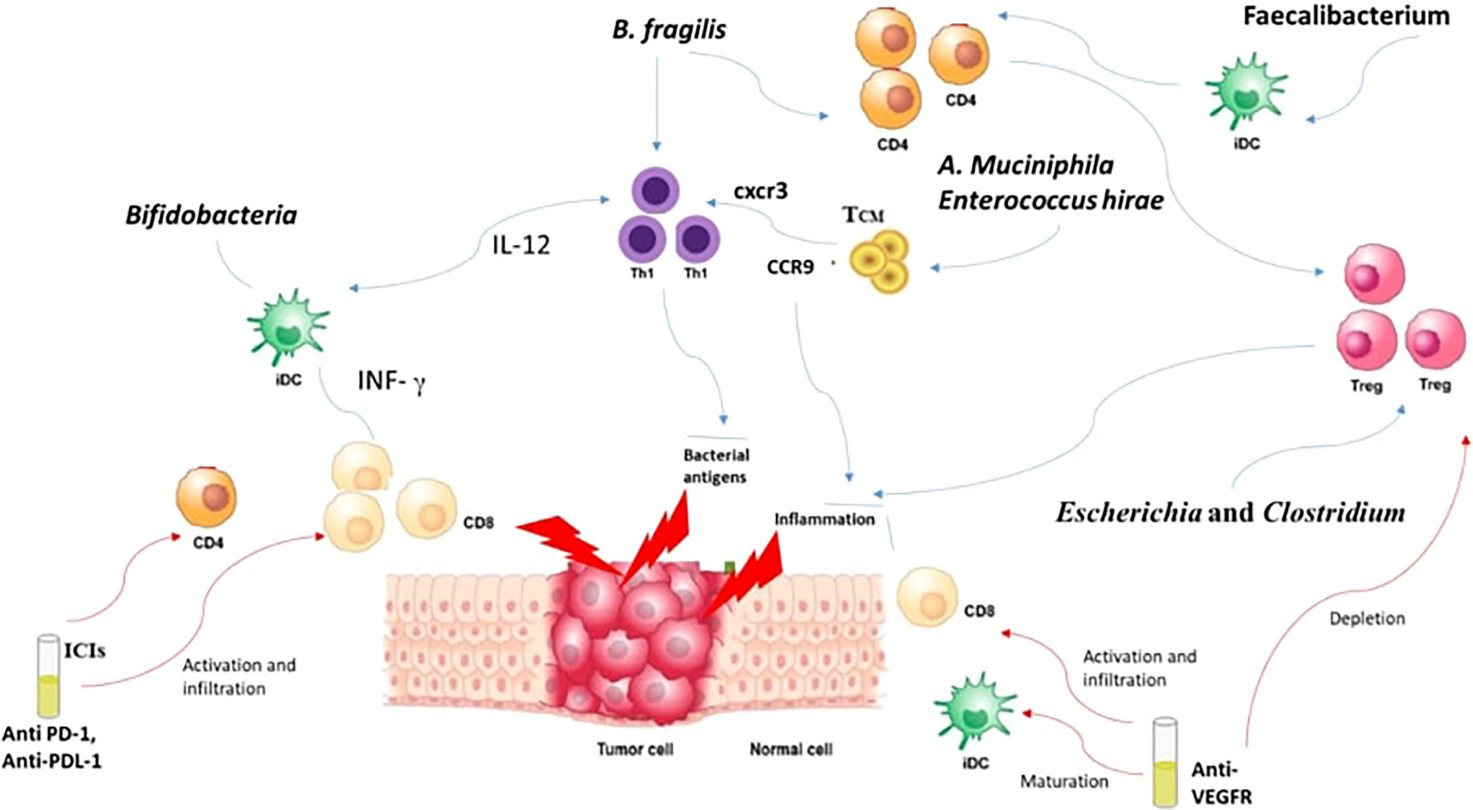
Interaction between microbiota, CPIs and anti-VEGFR.

GM is involved in the formation of different metabolites:

1. Trimethylamine N oxide (TMAO), a result of the oxidation of the trimethylamine (TMA), is released from the digestion of dietary TMA (163). TMAO is pro-atherogenic, it increases platelets aggregation and consequently increases the risk of thrombosis and strokes (164).
2. Uremic toxins are the results of amino acid breakdown by the GM. In addition, toxins as indoxyl sulfate, indoxyl glucuronide, indoleacetic acid, p-cresyl sulfate, p-cresyl glucuronide, phenyl sulfate, phenyl glucuronide, phenylacetic acid, and hippuric acid form through aromatic amino acid breakdown by the GM (165). In ECs, indoxyl sulfate activates NF-κB signaling pathway, upregulating ICAM-1 and monocyte chemotactic protein-1 (MCP-1) (166). In addition, indoxyl sulfate inhibits Nitric oxide synthesis and up-regulates reactive oxygen species (ROS), thereby contributing to endothelial dysfunction and atherosclerosis (167).
3. SCFA are results of bacterial, mainly *Lactobacillus* and *Bifidobacterium*, fermentation of carbohydrates (168). They are known for their role in increasing blood pressure. A byproduct of SCFA synthesis is butyric acid, produced through two different mechanisms: A- using the enzymes phosphotransbutyrylase and butyrate kinase (e.g., *Coprococcus* species) in order to convert butyryl-CoA into butyrate, B- butyryl-CoA/acetate CoA-transferase (e.g., *Faecalibacterium*, *Eubacterium*, and *Roseburia*) tend to convert butyryl-CoA into butyric acid (169–171). Sodium butyrate is also investigated for its influence on angiogenesis (172). Low levels of sodium butyrate stimulate angiogenesis and it stimulate up regulation of VEGFR and consequently the post receptor signaling pathway (172, 173). Thus, regulation of SCFA metabolites, such as butyrate and propionate, alter the host immune system through inducing the differentiation of Tregs (**Figure 2**) (174). VEGFR hence stimulate the angiogenesis of tumoral cells as well and here comes the role of anti-VEGFR in enhancing the immune system through the up regulation of CD8 (**Figure 2**) (174).
4. Gaseous metabolites include:
  4.1- Hydrogen Sulfide (H2S): Multiple studies have proven the role of H2S in the regulation of the circulatory system (175). In our gut, the dominant bacteria as *Desulfovibrio (D. piger, D. desulfuricans)*, *Desulfobacter*, *Desulfobulbus*, and *Desulfotomaculum* function as sulfate reducing agents. They produce H2S through a non-enzymatic course by using two substrates: a sulfate and an electron donor for the sulfate reduction (175). Other enzymatic reactions can occur through cysteine desulfhydrase causing the conversion of cysteine into H2S, pyruvate, and ammonia through several anaerobic bacterial strains (*E. coli, Salmonella enterica, Clostridia*, and *Enterobacter aerogenes*) (175). A 3^rd^ technique in H2S production is through the sulfite reduction that can take place in the presence of *E. coli*, *Salmonella*, *Enterobacter*, *Klebsiella*, *Bacillus*, *Staphylococcus*, *Corynebacterium*, and *Rhodococcus* (175). H2S highly contributes to the vasodilation of the vessels, therefore to the decrease of blood pressure and maintenance of homeostasis (176).
  4.2- Nitric oxide (NO): GM bacteria like *Lactobacillus* and *Bifidobacterium* help in the production of NO. Other bacteria like *Desulfovibrio vulgaris* converts it into nitrates. NO is important in vasodilation, but elevated concentrations of NO, produced from induction of the inducible isoform iNOS, can react with oxygen radicals, consequently causing further deterioration in infection-related conditions such as in septic shock or hypotension (177).
  4.3- Carbone monoxide (CO): Produced through the breakdown of heme with the help of the enzyme heme oxygenase (HO) into biliverdin, ferrous iron, and CO. We have two types of HO, one mainly expressed by the Gut mucosa, which is the inducible HO (HO-1) and the second known as constitutive HO (HO-2) (178). Similarly, CO exerts a vasodilative effect on ECs leading to cardiac protection (179).
5. Xenobiotic metabolites: GM contributes to the xenobiotic transformation. Polyphenols are diet components that represent examples of xenobiotic. They are mainly found in plants and algae, and used in cardiac medications. In the human body, they are metabolized by *Actinobacterium eggerthella lenta* that is responsible for its buffering, maintaining the safety of the body (180). Anthocyanins and phytoestrogens are two examples of xenobiotic metabolites. They both have protective effects against different pathological conditions, especially CVD and malignancies (181). Anthocyanins can positively alter the GM profiles, either through inducing the proliferation of good bacteria such as *Bifidobaterium* and *Lactobacillus*, or by inhibiting the growth of harmful bacteria like *Clostridium histolyticum* (182).

#### GM and Angiogenesis and CPIs

As discussed above, GM profiles became a key modulator of CPI responses. Moreover, GM also provides to the angiogenesis process as well to the vasculature development (183). For instance, VEGF was shown to be activated by bacterial polysaccharides, therefore enhancing the angiogenesis process (66). In addition, the bacteria present in tumor cells alter the vascular barrier of the gut, thus permitting the transfer of the GM into the circulation (66). This transfer or dislocation of the microbiota or the tumor bacteria contributes to the formation of the required environment and consequently enhancing metastasis (184). Tumors manage to grow through stimulating the budding of vessels from the surrounding vasculature (185). Different substrates are recognized as pro-angiogenic, specifically the VEGF-A, which helped in understanding the mechanisms that support tumor growth (186). Accordingly, the vasculature of the tumor is considered a target in the management of malignancies, and the most commonly used pathway is the anti-VEGF or the blockage of its receptors (187). Multiple trials have provided to the idea that anti-angiogenic treatment can stimulate the tumor immune response and at same time, the immune system can promote angiogenesis (187, 188). Here came the idea of combining CPI with anti-VEGF therapy to promote vascular normalization, where the immunosuppressive niche of the TME can be transformed into an immune stimulatory media supporting the entrance of the immune effector cells and their accumulation, leading to an enhanced anti-tumor activity by promoting hypoxia and inhibiting the function of the suppressive cells (**Figure 2**) (189).

Furthermore, angiogenesis was reported as an element influencing the response to CPIs. In fact, the combination of PDL-1 inhibitors and anti-VEGF showed an increase in PFS and OS in unrespectable HCC hepatocellular carcinoma and metastatic renal cell carcinoma (190, 191). This proves the complex relationship between the three, GM, angiogenesis and CPI response. Therefore, the combination of CPIs and anti-angiogenic therapy with manipulation of GM can be a therapeutic approach in the future holding promising results and augmenting CPIs response.

## Conclusion

In conclusion, Human microbiome, GM specifically, is a key effector in the process of metastasis. Through bacterial translocation, cancer cells from the initial tumor site exit the tissues to the systemic circulation, to disseminate to distant sites and invade different organs. Moreover, GM were shown to influence cancer progression through different mechanisms by exerting changes on the vasculature conformation, leading to an either accelerated or controlled metastatic process. Additionally, the alteration of GM has been proven to influence the response to CPIs, which is why it is crucial to further investigate the complex therapy combining GM alteration, in addition to anti-angiogenic therapy and CPIs for the ultimate goal of limiting cancer progression and metastasis early in the disease.

## Author Contributions

Conception or design of the work: AA, YB, and AS. Drafting the work: AA, YB, GA, YH, and AL. Critical revision for important intellectual content: AS. Approval of the version to be published: AA, YB, GA, YH, AL, and AS. Agreement to be accountable for all aspects of the work in ensuring that questions related to the accuracy or integrity of any part of the work are appropriately investigated and resolved: AA, YB, GA, YH, AL, and AS. All authors have read and agreed to the published version of the manuscript.

## Conflict of Interest

The authors declare that the research was conducted in the absence of any commercial or financial relationships that could be construed as a potential conflict of interest.

## Publisher’s Note

All claims expressed in this article are solely those of the authors and do not necessarily represent those of their affiliated organizations, or those of the publisher, the editors and the reviewers. Any product that may be evaluated in this article, or claim that may be made by its manufacturer, is not guaranteed or endorsed by the publisher.
